# Primary amenorrhea in adolescent girls: normal coitus or not? Always take a look in the physician's office

**DOI:** 10.1186/1472-6874-14-23

**Published:** 2014-02-10

**Authors:** Flora Bacopoulou, George Creatsas, George P Chrousos, Nikoleta Papanikolaou, Efthimios Deligeoroglou

**Affiliations:** 1 First Department of Pediatrics, Center for Adolescent Medicine and UNESCO Chair in Adolescent Medicine and Health Care, University of Athens Medical School, ‘Aghia Sophia’ Children's Hospital, 3 Thivon Street, Athens 115 27, Greece; 2 Division of Pediatric-Adolescent Gynecology and Reconstructive Surgery, Second Department of Obstetrics and Gynecology, University of Athens Medical School, ‘Aretaieion’ Hospital, Athens, Greece

**Keywords:** Amenorrhea, Adolescent health, Adolescent gynecology, Preventive health care visits, Urethral coitus, Müllerian agenesis, Inguinal hernia, Prolactinoma

## Abstract

**Background:**

Primary care physicians are frequently faced with the challenge of evaluating primary amenorrhea in adolescent girls. Approximately 15% of these women have abnormal genital examination, with Müllerian agenesis being the second most frequent cause. We report two cases of adolescents with Müllerian agenesis that presented to a tertiary adolescent medicine center with primary amenorrhea and the very rare sexual phenomenon of urethral coitus. The aim of this report is to emphasize the importance of performing a genital examination in girls who present with amenorrhea in the primary care setting, even if ‘normal’ vaginal sexual activity is assumed.

**Case presentations:**

A 19-year-old Caucasian and a 16-year-old Filipino girl presented to a tertiary adolescent medicine center with primary amenorrhea and a history of ‘normal’ vaginal coitus. Investigation revealed Müllerian agenesis in association with urethral coitus in both cases; neither patient suffered significant urethral damage to require urethra reconstruction. However, the first adolescent had recurrent pyelonephritis and renal scarring and the second had dysuria.

To the best of our knowledge, Case 1 also represents the second reported case of pituitary prolactinoma in association with Müllerian agenesis. The first adolescent underwent a hernia repair and vaginoplasty, whereas the second had vaginal dilatations.

**Conclusion:**

Our cases highlight the need for careful assessment of the external genitalia and vagina patency in all girls with amenorrhea, even if they report ‘normal’ vaginal sexual activity. Early identification of anatomic disorders such as Müllerian agenesis, will allow provision of proper care according to the patient’s needs and the existing abnormalities, and prevention of rare, unintentional but potentially physically and emotionally harmful, patterns of sexual intercourse.

## Background

Adolescent menstrual disorders are the most common gynecological complaint requiring the physician’s attention. Primary amenorrhea is the failure to menstruate by age 15 in the presence of normal secondary sexual development [1]. A first line pregnancy test, a thorough history, a complete physical examination and measurement of follicle stimulating hormone, thyroid stimulating hormone and prolactin, help identify the cause of amenorrhea in most cases [1]. In women with primary amenorrhea, approximately 15% have abnormal genital examination [1], with Müllerian agenesis being the second most frequent cause (10%). It is a congenital aplasia of the uterus and the upper part (2/3) of the vagina; type I is characterized by isolated utero-vaginal aplasia and type II, is associated with renal, skeletal abnormalities, inguinal ovarian hernias [2] and other defects. It has also been associated with a pituitary macroadenoma on one occasion [3]. In rare cases, Müllerian agenesis has also been associated with the sexual phenomenon of urethral coitus [4-7].

We describe two adolescent girls who presented to a tertiary adolescent medicine center with primary amenorrhea and ‘normal’ vaginal coitus and were subsequently found to have Müllerian agenesis in association with urethral coitus.

## Case presentations

### Case 1

**Figure 1:**
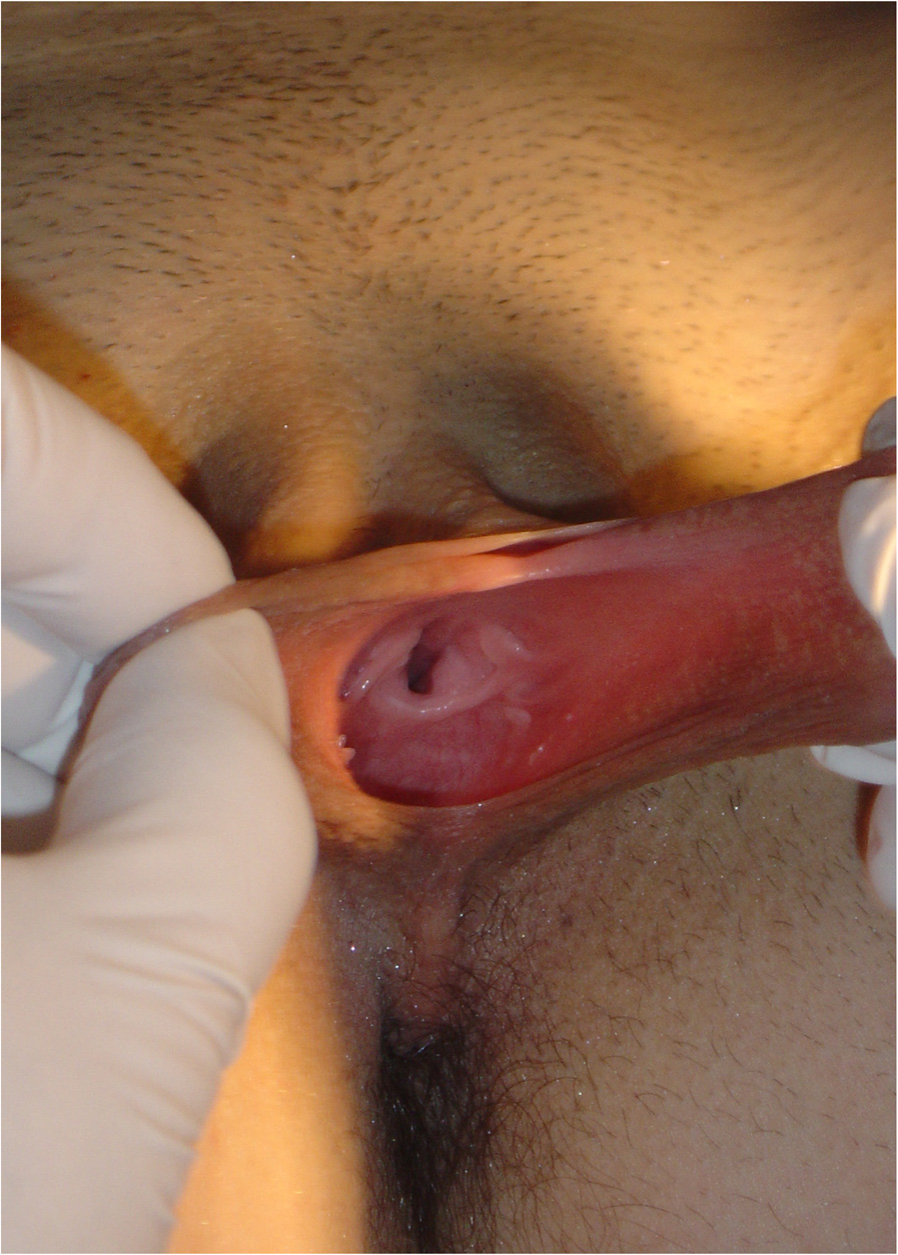
**Adolescent external genitalia in case 1.** Absent vagina, hypertrophic labia minora and wide urethral opening.

A 19-year-old Caucasian girl presented to us with primary amenorrhea. On questioning she reported ‘normal’ vaginal sexual activity and ‘normal’ results on ‘urethral Papanicolaou tests’ performed by her family physician. She had a past medical history of recurrent pyelonephritis but no complaints suggestive of urinary tract infection. Her family history was unremarkable. Physical examination showed an apparently normal female with normal secondary sexual characteristics. An inguinal mass was palpated on the left side. Labia majora and clitoris were normal, labia minora were hypertrophic and vagina was absent. External urethral meatus was dilated approximately 2.5 cm in diameter (Figure 1). Rectal examination did not reveal any evidence of cervix or uterus.

Abdominal ultrasound showed the left gonad present at the level of the inguinal canal. Both gonads had normal ovarian structure, volume and several follicles. No uterus was identified. There was scarring in the smaller, 8.9 cm left kidney. Serum renal function tests, urine analysis and culture were normal.

Results of hormonal testing were normal, except for prolactin which was elevated at 109 ng/dl. Tumor markers for ovarian cancer of the herniated gonad (lactate dehydrogenase, human chorionic gonadotropin, alpha-fetoprotein, cancer antigen CA 125) were also normal. Chromosomal analysis revealed a normal 46,XX female karyotype. Intravenous pyelography showed right ureteral stenosis and mild hydronephrosis. Cystourethroscopy showed that the urethra meatus was dilated but bore no other significant damage. Pituitary magnetic resonance imaging (MRI) revealed the presence of microadenoma (lateral oblique diameter of 9.5 mm and longitudinal diameter of 7.1 mm).

The patient subsequently underwent diagnostic laparoscopy that showed absence of a uterus, presence of the right fallopian tube and ovary in the right ovarian fossa and two thirds of the left ovary and fallopian tube in the left inguinal canal. Biopsy of both the gonads revealed typical ovarian tissue.

The patient was diagnosed with Müllerian agenesis. She underwent surgical reduction of the gonad into the pelvic cavity, repositioning of the fallopian tube and ovary and repair of the hernia, to decrease the risk of ovarian torsion and loss of ovarian function.

The patient decided to undergo surgical intervention for creation of a functional vagina. After careful psychologic preparation she was successfully managed surgically with the Creatsas modification of Williams vaginoplasty [8].

She was started on oral quinagolide (25 micrograms per day at bedtime for the first three days, followed by 50 micrograms per day for further three days and continued with a daily maintenance dose of 75 micrograms) aiming for full cure of her prolactinoma. After two years the pituitary adenoma is undetectable on MRI and serum prolactin has reverted to normal levels 9 ng/ml.

### Case 2

**Figure 2:**
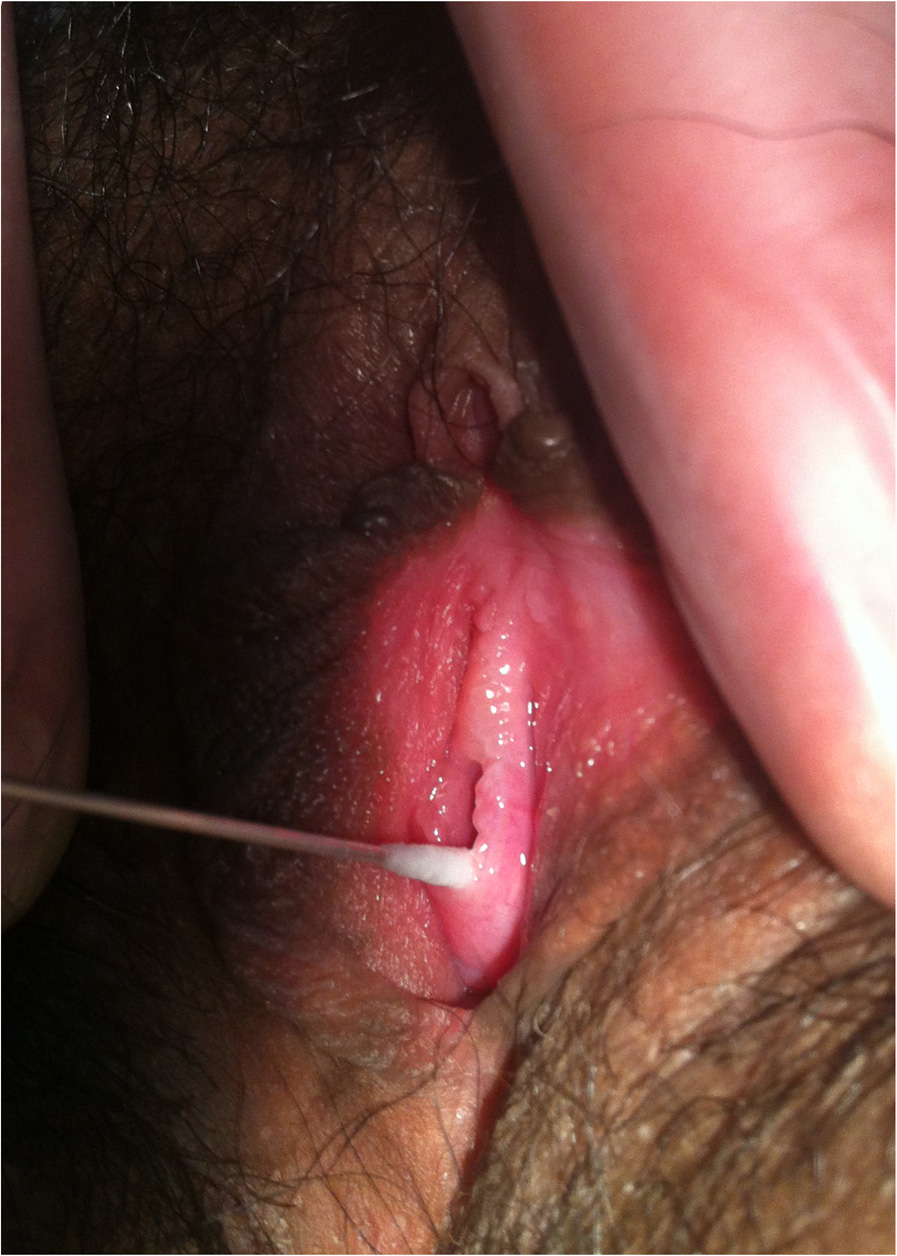
**Adolescent external genitalia in case 2.** Absent vagina, dilated external urethral meatus and everted urethral mucosa.

A 16-year-old Filipino girl presented to us with acute dysuria. She reported no menarche and intermittent abdominal pain. Her family history was unremarkable. On examination, she had normally developed secondary sexual characteristics, normal labia majora, labia minora and clitoris and a vaginal dimple. External urethral meatus was dilated with a maximum diameter of approximately 1.2 cm and the urethral mucosa was everted from the external meatus (Figure 2). Rectal examination did not reveal any evidence of cervix or uterus.

A detailed history revealed that dysuria had followed attempted sexual intercourse with penetration; the additional finding of a dilated urethra, helped to confirm urethral coitus. Hormonal investigation was normal, as well as the result of chromosomal analysis, consistent with a normal 46,XX female. Urine analysis and culture showed no evidence of infection. The abdominal ultrasound failed to visualize the uterus, whereas the ovaries and the renal system appeared normal. The abdominal MRI confirmed the sonographic findings showing no presence of uterus, cervix or vagina and normal appearing ovaries. Cystourethroscopy demonstrated no further abnormalities. The diagnosis of Müllerian agenesis was made and the adolescent expressed the desire for nonsurgical management; she was advised to manually place successive dilators on the vaginal dimple for 30 minutes to 2 hours per day. She was offered support by a psychologist.

## Discussion

Urethral coitus, is a very rare sexual phenomenon; 31 cases in the literature have been described, most commonly in women with vaginal abnormalities and only in four adolescents [4-7,9-11]. Complications include urethral dilatation, trauma, urinary incontinence and recurrent urinary tract infections; surprisingly, in some cases there are no associated complications.

We report here two cases of adolescents with Müllerian agenesis that presented with urethral coitus. Both of our patients were symptomatic; the first had recurrent pyelonephritis and renal scarring and the second had dysuria. Neither patient suffered significant urethral damage to require urethra reconstruction.

We assume that in patients with Müllerian agenesis the urethra can be dilated relatively easily, as the absence of the vagina weakens the urethral support system and allows penetration during intercourse via the urethral orifice. It was striking that in the first case, the adolescent had been followed for years by her family physician and, surprisingly, she reported ‘normal’ results on ‘urethral Papanicolaou tests’.

To the best of our knowledge, Case 1 also represents the second reported case of pituitary prolactinoma in association with Müllerian agenesis. Although both conditions are rare, most likely the concurrence is coincidental. When the defective genes for Müllerian agenesis are known [12], a possible conjecture may be then formulated.

## Conclusions

Our cases highlight the need for careful assessment of the external genitalia and vagina patency in all girls with amenorrhea, even if they report ‘normal’ vaginal sexual activity. The American Academy of Pediatrics promotes the inclusion of the external gynecologic examination in the primary care setting as part of the annual comprehensive physical examination of children and adolescents of all ages and a complete pelvic examination for adolescents with amenorrhea [13].

Early identification of disorders such as Müllerian agenesis, will allow provision of proper care according to the patient’s needs and the existing abnormalities, and prevention of rare, unintentional but potentially physically and emotionally harmful, patterns of sexual intercourse.

### Consent

Written informed consent was obtained from each patient for publication of these Case reports and the accompanying images. A copy of both written consents is available for review by the Editor of this journal.

## Abbreviations

MRI: Magnetic resonance imaging.

## Competing interests

The authors declare that they have no financial or non-financial competing interests in relation to this manuscript.

## Authors’ contributions

All authors have made substantial contributions to conception, acquisition and interpretation of data. They have participated in drafting the article and they have approved the final submitted version. FB acquired and interpreted the data, conceptualized and drafted the initial manuscript, and approved the final submitted version. GC reviewed and interpreted the data, revised the manuscript, and approved the final manuscript as submitted. GPC reviewed and interpreted the data, revised the manuscript, and approved the final manuscript as submitted. NP actively participated in data collection, in drafting the manuscript, and approved the final manuscript as submitted. ED supervised data collection and interpreted the data, critically reviewed the manuscript, and approved the final manuscript as submitted.

## Authors’ information

Dr FB (MD, PhD) is a Pediatrician in adolescent medicine who has been trained for two years in Pediatric Adolescent Gynecology and has completed an International Fellowship in Pediatric and Adolescent Gynecology (IFEPAG) under the auspices of the International Federation of Infantile and Juvenile Gynaecology (FIGIJ). She is a research associate at the Center for Adolescent Medicine (C.A.M.), a tertiary referral center for adolescents and young people in Greece, that hosts the UNESCO Chair in Adolescent Medicine and Health Care. Professor GPC (MD, MACP, MACE, FRCP London), the Chairholder and Director of C.A.M. and of the First Department of Pediatrics of the Athens University, is a Professor in Pediatric Endocrinology.

Professor in Obstetrics and Gynecology GC (MD, FACS, FACOG, FRCOG), is the Director of the Division of Pediatric-Adolescent Gynecology and Reconstructive Surgery and of the Second Department of Obstetrics and Gynecology of the Athens University. Professor ED (MD, PhD) is an Associate Professor in Obstetrics and Gynecology of the Division of Pediatric-Adolescent Gynecology and Reconstructive Surgery, that is the only tertiary referral center for women with Mayer-Rokitansky-Küster-Hauser syndrome in Greece. He is Vice President of the International Federation of Infantile and Juvenile Gynaecology (FIGIJ).

## Pre-publication history

The pre-publication history for this paper can be accessed here:

http://www.biomedcentral.com/1472-6874/14/23/prepub
